# Integrating Nonindividual Patient Features in Machine Learning Models of Hospital-Onset Bacteremia

**DOI:** 10.1001/jamanetworkopen.2025.18815

**Published:** 2025-07-02

**Authors:** M. Cristina Vazquez Guillamet, Jingwen Zhang, Alice Bewley, Andrew Atkinson, Heidi Holtz, Ziqian Wang, Nicole Brougham, Chenyang Lu, Marin H. Kollef, Philip Payne, David K. Warren, Victoria J. Fraser

**Affiliations:** 1 Division of Infectious Diseases, Department of Medicine, Washington University School of Medicine, St Louis, Missouri; 2 Division of Pulmonary and Critical Care Medicine, Department of Medicine, Washington University School of Medicine, St Louis, Missouri; 3 McKelvey School of Engineering, Department of Computer Science & Engineering, Washington University, St Louis, Missouri; 4 Department of Research, Goldfarb School of Nursing at Barnes-Jewish College, St Louis, Missouri; 5 Center for Practice Excellence, Goldfarb School of Nursing at Barnes-Jewish College, St Louis, Missouri; 6 Department of Medicine, Washington University School of Medicine, St Louis, Missouri

## Abstract

**Importance:**

Hospital-onset bacteremia and fungemia (HOB) are common and potentially preventable complications of hospital care.

**Objective:**

To assess whether nonindividual patient features, which summarize interactions with other patients and health care workers (HCWs), can contribute to predictive and causal machine learning models for HOB.

**Design, Setting, and Participants:**

This prognostic study included adult patients admitted to Barnes-Jewish Hospital, an academic hospital in St Louis, Missouri, in 2021. Analyses were developed between October 2023 and August 2024 and in April 2025.

**Exposure:**

Individual patient features were extracted from electronic health records and used to engineer nonpatient features, including interactions with HCWs and direct or indirect (consecutive room occupancy) patient contact.

**Main Outcomes and Measures:**

HOB was defined as a positive blood culture after the third day of hospitalization. Patients who were hospitalized for more than 3 days were considered at risk for the outcome. We developed 3 gradient boosting models: 2 predictive (with patient features only and with both patient and nonpatient features to predict the occurrence of HOB) and 1 causal to test the association of nonpatient features and HOB. Predictive performance is reported using area under the receiver operating characteristic curve (AUROC) and area under the precision-recall curve (AUPRC), and the results of the causal model are reported as difference in average effects. Sensitivity analyses separated intensive care unit–onset and ward-onset HOB and included a methicillin-resistant *Staphylococcus aureus*–specific model to adjust for colonization pressure.

**Results:**

Among the 52 442 patients, 34 855 (66.5%) had admissions longer than 72 hours and were included for analysis; of these, 556 (1.6%) developed HOB. The median age for the included patients was 60 (IQR, 44-70) years, 50.5% were female, and obesity was the most frequent comorbidity (25.0%). Nonpatient features, such as a prior occupant of the same room receiving antipseudomonal beta-lactams and the mean number of HCWs per day for the 7 days preceding HOB, improved the model’s performance (AUROC, 0.88 [95% CI, 0.88-0.89]; AUPRC, 0.20 [95% CI, 0.20-0.22]) compared with the patient-only model (AUROC, 0.85 [95% CI, 0.85-0.86]; AUPRC, 0.13 [95% CI, 0.12-0.14]) (*P* < .001). These 2 features were also associated with a higher likelihood of HOB in the causal gradient boosting model.

**Conclusions and Relevance:**

These findings suggest that nonindividual patient features may contribute to a comprehensive analysis of HOB when integrated with individual patient features in a machine learning model.

## Key Points

**Question:**

Do nonpatient features that summarize patient interactions with other patients and health care workers impact machine learning models for hospital-onset bacteremia?

**Findings:**

This prognostic study of 52 442 adult patients hospitalized during 2021 found that nonindividual patient features contributed to causal machine learning models and improved predictive model performance.

**Meaning:**

These findings suggest that machine learning models can integrate multifactorial interactions between individual and nonindividual risk factors for a comprehensive analysis of hospital-onset bacteremia.

## Teaser

This prognostic study assesses whether nonindividual patient features that summarize interactions with other patients and health care workers can contribute to machine learning models to predict hospital-onset bacteremia (HOB).

## Introduction

Hospital-onset bacteremia and fungemia, collectively referred to as HOB, are common and often preventable complications of hospital care, associated with high mortality and morbidity rates as well as significant secondary costs.^1,2,3,4,5^ HOB includes both central line–associated bloodstream infections (CLABSIs) and non-CLABSIs, with the burden of cases without central lines being 4 times higher, leading to increased overall mortality. In addition to improving central venous catheter (CVC) care, studies have primarily focused on patient-related characteristics, such as demographics and comorbidities^6,7,8,9,10^; however, these factors are nonmodifiable and offer limited opportunities for prevention efforts.

HOB occurs within the complex setting of a hospital. Investigating nonpatient variables could improve the ability to determine which patients will develop HOB and identify actionable targets for prevention, particularly since up to two-thirds of HOB cases may be preventable.^1,2^ We envision the hospital as a dynamic network where patient risk is influenced not only by their individual characteristics but also by their locations within the hospital and their interactions with other patients through health care workers (HCWs) and the hospital environment. Recent studies have shown that occupying hospital rooms previously inhabited by patients with certain microbes or exposure to antibiotics can increase the risk of acquiring antibiotic-resistant organisms (AROs) and hospital-acquired infections (HAIs).^11,12,13,14^ Nursing variables have also been associated with a higher risk of HAIs.^15^ In this study, we leveraged multimodal electronic health record (EHR) data sources as input features for machine learning algorithms with feature engineering. We examined both patient-related and non–patient-related features in predictive and causal models to identify relevant features associated with HOB in a large cohort of adult patients admitted to Barnes-Jewish Hospital (BJH), a tertiary academic hospital in St Louis with 1269 beds, in 2021.

## Methods

### Study Population and Data Source

For this prognostic study, a retrospective cohort analysis was performed at Washington University/BJH and conducted according to the Washington University Institutional Review Board Protocol, with the need for informed consent waived due to the noninterventional, retrospective nature of the study. All adult patients admitted to BJH between January 1 and December 31, 2021, were included. Analyses were developed between October 2023 and August 2024 and in April 2025. Data were directly extracted and timestamped as recorded in the Epic EHR (Epic Systems Corporation).

### Outcomes

HOB was defined as the growth of microorganisms from a blood culture obtained on the third calendar day or later of hospitalization and not present at admission. We labeled the microorganisms according to the National Healthcare Safety Network list of true pathogens and contaminants.^16^ Only patients who were hospitalized for more than 3 days were considered at risk for the outcome. The first episode of HOB during each hospitalization was included in the analyses.

### Feature Selection, Engineering, and Data Processing

The full list of features and preprocessing details are presented in eTable 1 in Supplement 1. All risk factors had to predate the blood culture collection by at least 48 hours.

Patient risk factors included demographics (age, sex), all comorbidities (defined by *International Statistical Classification of Diseases and Related Health Problems, Tenth Revision* codes), procedures (endotracheal intubation, CVC placement), severity of illness (mechanical ventilation and shock requiring vasopressor or inotropic support), and antibiotics administered prior to the HOB episode. We created 3 separate binary features to indicate the use of different antibiotic classes: beta-lactams, antipseudomonal beta-lactams, and others.

Nonpatient risk factors were created to quantify the interactions between the patient and the hospital environment, HCWs, and other patients. We included duration of hospitalization in each room (intensive care units [ICUs] and wards) and wait times in the emergency department (ED) prior to hospital admission. Nonpatient features included sharing a room with a different patient (for at least 24 hours in double occupancy rooms), inhabiting the same room as prior patients for more than 24 hours, and the mean number of interactions with HCWs per day for the 7 days preceding HOB. We focused on physicians (attendings and all levels of trainees) and nursing staff. We extracted the HCW information from clinical notes in the EHR. At BJH, physicians and nurses are mandated to write notes on each patient at least daily. For patients who did not develop HOB, we performed the same calculations accounting for the 7 days before discharge. We chose 7 days for HOB because it most likely has a shorter timeframe between exposure and infection than the 14 days used for *Clostridium difficile* infections. We collected culture data and administered antibiotics for all patients and classified indirect antibiotic exposure if prior occupants received any or all of the antibiotic classes.

### Training and Testing Machine Learning Models

We developed 3 gradient-boosting models: 2 exploratory predictive models (one based on patient features only and the second model incorporating both patient and nonpatient features to predict the occurrence of HOB) and a causal model to test the relevant nonpatient features identified by the predictive models. Extreme gradient boosting (XGBoost) builds decision trees sequentially by randomly sampling the patients and features, with each iteration learning from the errors of the previous ones.^17^ Causal XGBoost estimates the average effect in 2 groups by using tree-based models and incorporating causal inference techniques from neural network–based models.^18^ We included all patient features, as well as the duration of stay in the ED and ICU and the overall hospital stay. We then estimated the effect difference for the nonpatient features identified in the predictive models. A difference greater than 0 indicates that the presence of the feature is associated with a higher likelihood of HOB. Model performance was evaluated using the area under the receiver operating characteristic curve (AUROC) and the area under the precision-recall curve (AUPRC). In a highly imbalanced dataset, AUPRC evaluates the mean positive predictive value over all sensitivity thresholds and measures the model’s ability to predict positive cases. While a higher AUPRC is generally considered superior, there is no defined cutoff value. Variable importance was ranked with Shapley additive explanations (SHAP).^19^ A positive SHAP value indicates that the feature increased the model’s ability to predict the studied outcome, whereas a negative SHAP value indicates that the feature lowered the association. The magnitude of the SHAP value reflects the feature’s contribution to the model’s predictive performance.

### Statistical Analysis

Due to the limited number of positive HOB cases, we employed 10-fold cross-validation to evaluate predictive performance. The entire dataset was divided into 10-folds using stratified K-fold cross-validation to maintain a similar positive case ratio across each fold. In each iteration, the model was trained on 9 folds and evaluated on the remaining 1 fold. The training data were further split into 80% for training and 20% for testing. Testing data were used for hyperparameter tuning and model selection. After completing 10 iterations, all patients were evaluated, and AUROC and AUPRC were computed to assess the model performance and compared using a *t* test. We present the mean, SD, and 95% CI of results across the 10 independent 10-fold cross-validation runs for each of the performance metrics. The 95% CIs were calculated by taking the 2.5th and 97.5th percentiles from the distribution of area under the curve values. For sensitivity analyses, we separated the model into ICU-onset and non-ICU HOB. To investigate possible microbe-specific in-hospital transmission, we developed a methicillin-resistant *Staphylococcus aureus* (MRSA)–specific predictive model. For this model, we added direct contact (sharing a room simultaneously) and indirect contact (occupying a room consecutively) with patients known to be colonized or infected with MRSA, including interactions with the same member of the health care team. Additionally, colonization pressure, defined as the proportion of patients with MRSA on the same ward in the 14 days preceding HOB, was included in the model.

The gradient-boosting model was implemented using XGBoost 1.7.7 and C-XGBoost, with tuned tree depth, learning rate, and number of estimators. The machine learning pipeline of training and evaluation was implemented on scikit-learn 1.2.2 and Python 3.8.18. We separated the predictive and causal machine learning analyses and report our results according to published recommendations for machine learning prediction models and the Strengthening the Reporting of Observational Studies in Epidemiology (STROBE) reporting guideline.^20,21,22,23^

## Results

A total of 52 442 patients were admitted to BJH in 2021, with 34 855 patients (66.46%; median age, 60 [IQR, 44-70] years; 50.5% female and 49.5% male) having admissions longer than 72 hours, putting them at risk for HOB. Among these, 556 patients (1.6%) developed HOB at a median duration of hospitalization of 11.2 (IQR, 6.4-20.9) days with a microbe not cultured at admission. The most isolated pathogens were *S aureus* (89 cases [16.0%], including 70 [78.7%] resistant to methicillin), *Klebsiella pneumoniae* (49 cases [8.8%], including 16 [32.7%] resistant to ceftriaxone), and Candida spp (59 cases [10.6%]). The most common sources were CLABSI (94 cases [16.9%]), pulmonary infections (53 cases [9.5%]), and urinary tract infections (23 cases [4.1%]). Comorbidities, including previous infections and exposure to antibiotics, were more common in patients with HOB (Table) who were also more critically ill. Patients with HOB were more likely to have a CVC, have an ICU stay, and receive antipseudomonal beta-lactams. Duration of hospitalization prior to HOB was longer in patients with HOB, and they had more daily contacts with HCWs compared with patients without HOB (Table). A mean of 7.0 (IQR, 5.4-8.8) physicians and nurses provided care daily to patients with HOB preceding the HOB episode, compared with a mean of 6.4 (IQR, 5.5-7.5) physicians and nurses for patients without HOB (*P* < .001).

**Table.** Cohort Description

Feature	No. (%)			*P* value
	Entire cohort (N = 34 855)	HOB (n = 556)	Non-HOB (n = 34 299)	
Demographics				
Age, median (IQR), y	60 (44-70)	60 (51-68)	60 (43-70)	.06
Sex				
Male	17 249 (49.5)	350 (62.9)	16 899 (49.3)	.001
Female	17 606 (50.5)	206 (37.1)	17 400 (50.7)	
Comorbidities				
End-stage kidney disease	1634 (4.7)	52 (9.4)	1582 (4.6)	.001
COPD, emphysema, chronic bronchitis	5680 (16.3)	66 (11.9)	5614 (16.4)	.01
Liver cirrhosis	1724 (4.9)	56 (10.1)	1668 (4.9)	.001
Hematological malignant neoplasms	2682 (7.7)	163 (29.3)	2519 (7.3)	.001
Solid organ transplant	1005 (2.9)	30 (5.4)	975 (2.8)	.001
Bacterial pneumonia	497 (1.4)	24 (4.3)	473 (1.4)	.001
History of sepsis	2664 (7.6)	128 (23)	2536 (7.4)	.001
GNB infections	1910 (5.5)	45 (8.1)	1865 (5.4)	.01
Resistance to antimicrobials	536 (1.5)	29 (5.2)	507 (1.5)	.001
Obesity	8722 (25.0)	249 (44.8)	8473 (24.7)	.001
Central venous catheter	1829 (5.3)	129 (23.2)	1700 (5.0)	.001
Severity of illness				
Septic shock	3952 (11.3)	167 (30)	3785 (11.0)	.001
Mechanical ventilation	876 (2.5)	31 (5.6)	845 (2.5)	.001
ICU stay prior to HOB	8323 (23.9)	287 (51.6)	8036 (23.4)	.001
Duration of ICU stay, median (IQR), h	0.0 (0.0-0.0)	0.5 (0.0-169.8)	0.0 (0.0-0.0)	.001
Duration of hospitalization prior to HOB, median (IQR), h	147.8 (101.2-250.4)	268.5 (152.9-501.6)	147 (101.0-246.7)	.001
Antibiotic use				
Beta-lactams	14 955 (42.9)	227 (40.8)	14 728 (42.9)	.34
Antipseudomonal beta-lactams	10 944 (31.4)	353 (63.4)	10 591 (30.9)	.001
Other antibiotics	14 955 (42.9)	312 (56.1)	14 643 (42.7)	.001
Hospital features				
Mean number of HCWs per day for the 7 d prior to HOB, mean (±2 SD)	6.4 (5.5-7.6)	7.0 (5.4-8.8)	6.4 (5.5-7.5)	.001
Number of rooms inhabited prior to HOB, median (IQR)	2.0 (2.0-3.0)	2.0 (1.0-3.0)	2.0 (2.0-3.0)	.001
ED wait times, median (IQR), h	4.4 (0.0-7.5)	2.9 (0.0-0.0)	4.4 (0.0-7.5)	.001
Direct and/or indirect interaction with other patients				
Duration of room sharing in the 7 d prior to HOB, median (IQR), h	33.5 (0.0-153.6)	0.0 (0.0-8.8)	36.6 (0.0-154.5)	.001
Shared rooms with patients taking antibiotics	12 260 (35.2)	119 (21.4)	12 141 (35.4)	.001
Inhabiting a room after a patient taking antibiotics	22 762 (65.3)	475 (85.4)	22 287 (65)	.001
Inhabiting a room after a patient taking antipseudomonal beta-lactams	12 251 (35.1)	366 (65.8)	11 885 (34.7)	.001

Abbreviations: COPD, chronic obstructive pulmonary disease; ED, emergency department; GNB, gram-negative bacteria; HCW, health care worker; HOB, hospital-onset bacteremia; ICU, intensive care unit.

**Figure 1.**
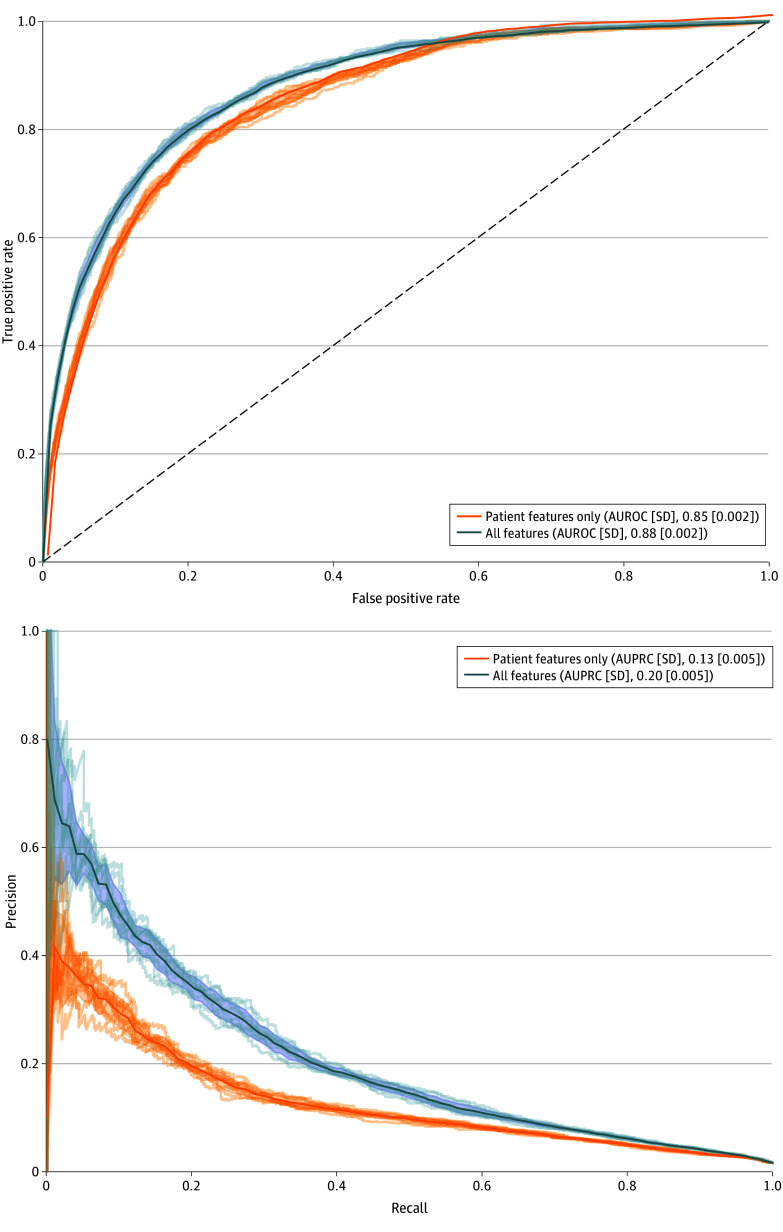
Predictive Performance for the 2 Gradient-Boosting Models (Patient Features Only vs Patient and Nonpatient Features) The dashed line in panel A indicates a random classifier or baseline performance. AUPRC indicates area under the precision-recall curve; AUROC, area under the receiver operating characteristic curve.

**Figure 2.**
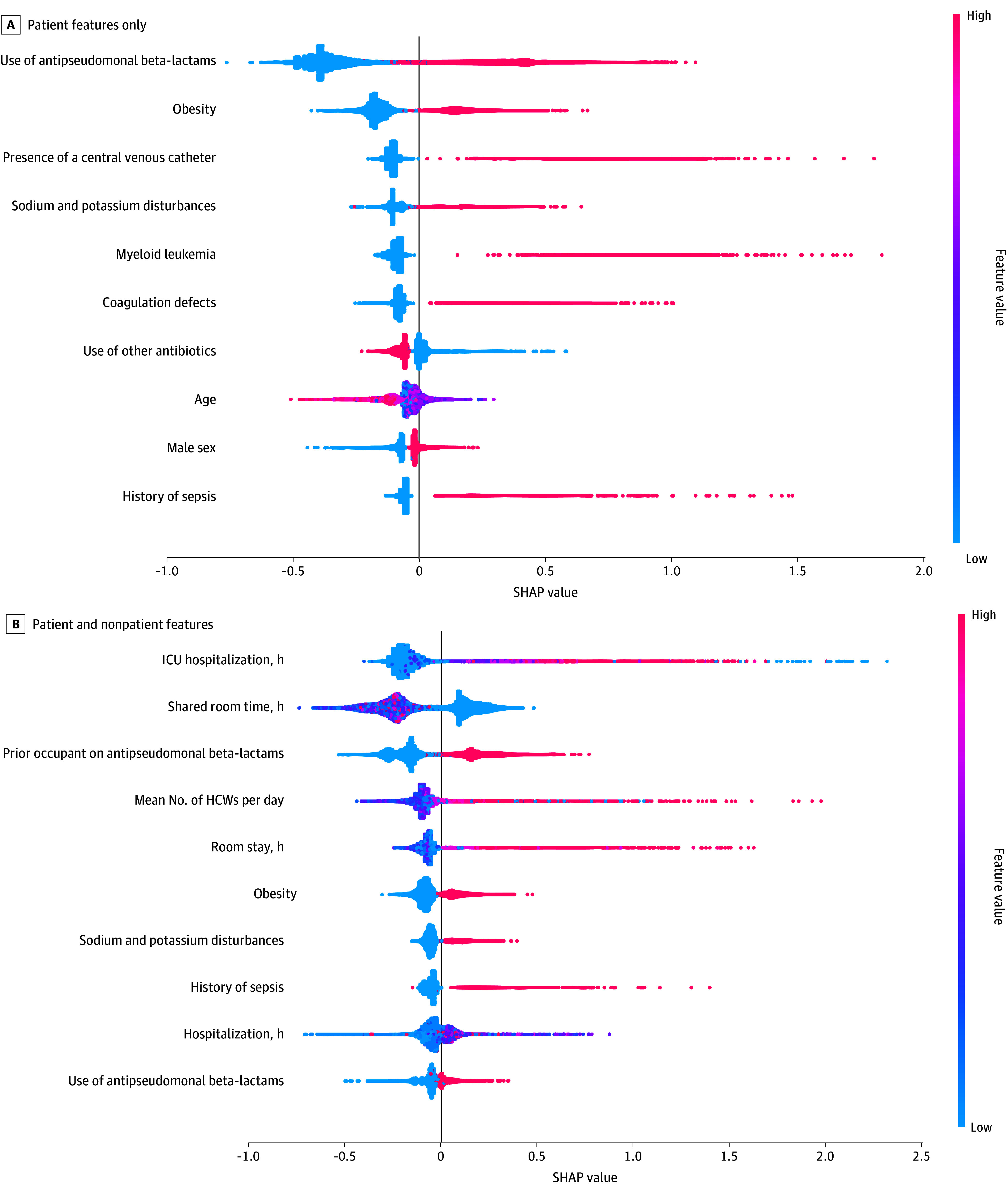
Top 10 Most Important Features for Gradient-Boosting Model Performance for Any Hospital-Onset Bacteremia and Fungemia HCW indicates health care worker; SHAP, Shapley additive explanations.

The predictive model incorporating both patient and nonpatient features had superior performance compared with the model based on patient features only (AUROC, 0.88 [95% CI, 0.88-0.89] and AUPRC, 0.20 [95% CI, 0.20-0.22] vs AUROC, 0.85 [95% CI, 0.85-0.86] and AUPRC, 0.13 [95% CI, 0.12-0.14]; *P* < .001 for AUROC and *P* < .001 for AUPRC) (Figure 1). The most important individual feature was use of antipseudomonal beta-lactams, while duration of ICU hospitalization prior to HOB was the most relevant feature in the predictive model with both patient and nonpatient risk factors. Administration of antipseudomonal beta-lactams, obesity, sodium and potassium disturbances, and history of sepsis were relevant in both models (Figure 2). Additional features, such as the duration of ICU stay, prior room occupant receiving antipseudomonal beta-lactams, mean number of HCWs per day for the 7 days preceding HOB, and the duration of hospitalization and room stay, were significant in the comprehensive model. In the causal models, the average effect difference for prior occupants receiving antipseudomonal beta-lactams after including all patient features and ED, ICU, and hospital length of stay was 0.4% (95% CI, 0.3%-0.5%). The average effect difference for interactions with HCWs as a binary variable was 0.7% (95% CI, 0.3%-0.5%).

**Figure 3.**
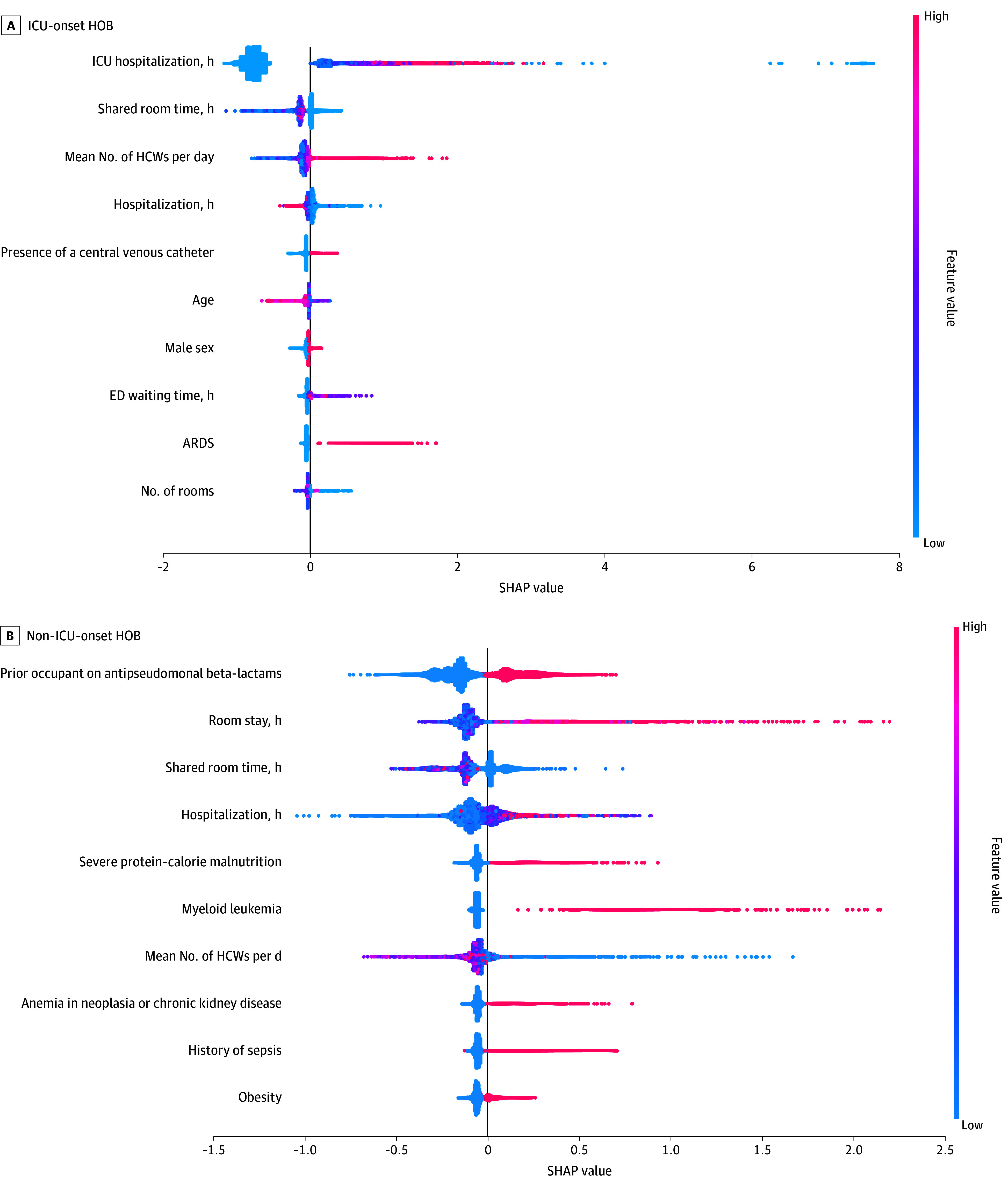
Top 10 Most Important Features for Gradient-Boosting Model Performance for ICU-Onset and Non-ICU–Onset HOB ARDS indicates acute respiratory distress syndrome; ED, emergency department; HCW, health care worker; HOB, hospital-onset bacteremia; ICU, intensive care unit; SHAP, Shapley additive explanations.

A total of 222 patients (0.64%) experienced ICU-onset HOB. The mean number of HCWs remained predictive in both ICU and non-ICU models (Figure 3). The ICU-onset HOB model was characterized by ICU-specific features, such as the duration of hospitalization in the ICU, presence of a CVC, and diagnosis of adult respiratory distress syndrome. Duration of hospitalization was predictive of HOB in non-ICU onset and was inversely correlated in ICU-onset HOB. A prior occupant receiving antipseudomonal beta-lactams was a predictor for HOB occurring on the wards.

**Figure 4.**
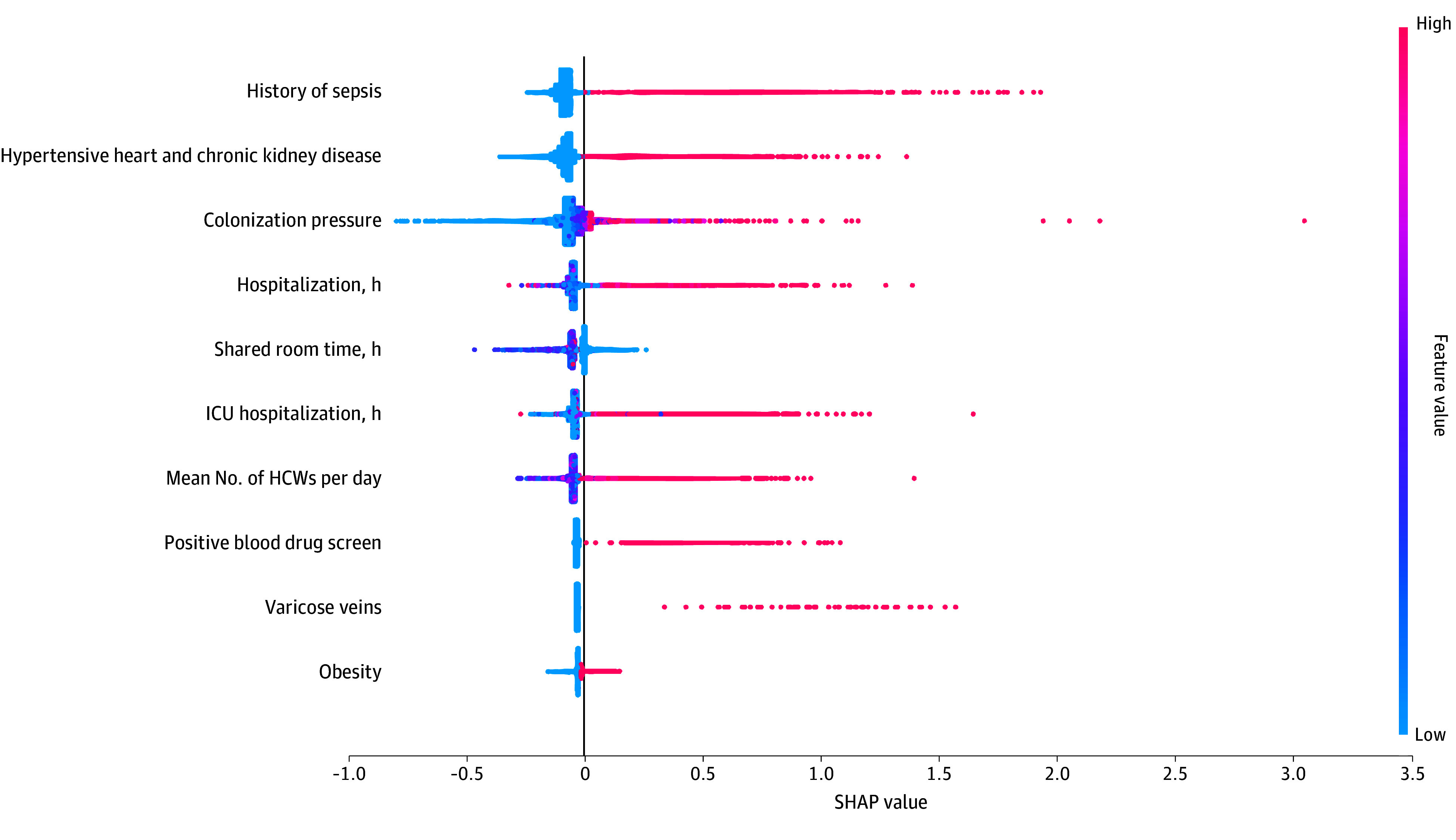
Top 10 Most Important Features for Gradient-Boosting Model Performance in MRSA HOB Model HCW indicates health care worker; HOB, hospital-onset bacteremia; ICU, intensive care unit; MRSA, methicillin-resistant *Staphylococcus aureus*; SHAP, Shapley additive explanations.

Seventy patients (0.2%) were diagnosed with MRSA HOB. Colonization pressure was higher in patients with MRSA HOB compared with patients without MRSA (eTable 2 in Supplement 1) and was predictive of MRSA HOB (Figure 4). The mean number of HCWs per day remained predictive, along with duration of ICU stay.

## Discussion

Our study analyzed data from a large and diverse cohort of patients admitted to a Midwest academic center in 2021. The data indicated that nonpatient risk features increased the predictive performance of HOB models, at times surpassing individual risk factors. Antipseudomonal antibiotic use was an important predictive feature both directly and indirectly. Interaction with HCWs and use of antipseudomonal beta-lactams in prior occupants were associated with higher risk of HOB in the causal models. Indwelling devices and CVC care are modifiable risk factors for bacteremia, and adherence to prevention bundles has significantly improved the rates of CLABSI.^24^ Hospitalized patients receive care within a complex network, involving direct and indirect contact with other patients, HCWs, and the hospital environment. The risk of HOB is likely a marker of contact with the hospital setting rather than being determined solely by individual patient features and exposure to indwelling devices. Machine learning is widely recognized for its ability to handle big data and improve predictions without necessarily establishing causality. However, recent gradient boosting models, such as causal-XGBoost, aim to estimate the effects of risk factors on outcomes. Our analysis leverages the computational power of XGBoost to explore nonpatient features that may predict and act as risk factors for HOB. The exact mechanism of how potential risk factors influence HOB should be investigated in future studies.

Recent literature has expanded our understanding of HAIs by placing patients within the hospital setting. Multiple studies have assessed the risk of acquiring AROs based on patients’ exposure to infected or colonized roommates or prior room occupants.^11,12,13,25,26,27^ These studies, designed as case-control or epidemiological studies, provided stronger evidence for the transmission of MRSA, vancomycin-resistant enterocococi, and *C difficile* by pairing bacterial isolates based on species identification and antimicrobial susceptibility testing. However, antimicrobial susceptibility testing has limitations in assessing microbial relatedness because genetically related bacteria may have different antibiograms, and distinct pathogens may share similar antibiograms. Additionally, these studies mainly relied on positive clinical culture, often neglecting the smaller but potentially relevant contribution of colonized patients to transmission. Jones et al^28^ included active surveillance studies conducted for MRSA across 112 Veterans Affairs hospitals to estimate the association of admission prevalence with acquisition rates as a surrogate for in-hospital transmission and positive nosocomial clinical cultures. Their analysis, which included 2.9 million surveillance tests from 1.4 million patient admissions, concluded that a 10% increase in a hospital’s MRSA admission prevalence was linked to a 9.7% increase in its weekly acquisition rate and a similar increase in weekly nosocomial culture rates. For MRSA-specific transmission, our predictive model also identified colonization pressure as a relevant predictor.

In addition to the clonal dissemination of AROs, interspecies plasmid-mediated transmission contributes to the dissemination of powerful AROs, such as carbapenem-resistant *Enterobacterales*.^29,30,31,32^ Recent studies have investigated the herd effect of antibiotic administration, expanding the understanding of how individual antibiotic use affects rates of *C difficile* and infections caused by AROs. Brown et al^33^ demonstrated that ward-level antibiotic prescribing was associated with a statistically significant and clinically relevant increase in the risk of *C difficile* infection in hospitalized patients, even after adjusting for patient and ward characteristics. In a study that included more than 100 000 pairs of patients, the receipt of antibiotics by prior bed occupants was associated with an increased risk of *C difficile* infection in subsequent patients.^14^ Our study contributes to the literature on the herd effect of antibiotics by linking the administration of antipseudomonal beta-lactams in prior occupants with an increased risk of HOB in subsequent patients occupying the same room. Our findings should be tested in microbe-specific studies ideally relying on whole-genome sequencing.

Nosocomial infections may be indicative of the quality of care delivered by hospitals. Patient-to-nursing ratios and understaffing have been linked to inferior quality of care.^34,35,36^ The Nurse Staffing Standards for Hospital Patient Safety and Quality Care Act of 2021 established protective standards to address these issues.^37^ Since BJH adheres to these standards, there was no significant variability in patient-to-nursing ratios in our cohort. The patient-to-nursing ratio, while important, lacks sensitivity in measuring workload. For instance, patients with the same medical acuity may have vastly different care needs depending on their level of independence. This limitation highlights the need for more nuanced metrics in evaluating the relevance of workload metrics as potential risk factors for HOB.^38^ Nursing burnout^39^ affects up to 50% of nursing staff and has been associated with nosocomial infections in a meta-analysis of 85 studies worldwide.^15^ One of the key findings from our study was that the mean number of daily HCWs interacting with the patients emerged as a relevant feature in both the predictive and causal models. This may be attributed to sicker patients requiring multiple consultants, but it also suggests an actionable marker for HCW organization. Prioritizing consistency in care (ie, ensuring the same nurses and physicians attend to the same patients) or having dedicated wards for patients with infections could enhance care delivery and potentially reduce infection risks.

### Limitations

This study has some limitations. We focused primarily on nurses and physicians, excluding other hospital staff who also provide care to patients. Intensity (eg, patient cleaning) and duration of contact may be valuable features, but they are not consistently or accurately documented in EHR data. At the time of the study, BJH had infection control policies that met or exceeded isolation and environmental cleaning protocols for AROs. However, we did not have access to data on how well these protocols were followed. Additionally, we did not include all procedures, nor did we account for the duration of CVC use. We did not categorize all possible antibiotics separately due to the vast number of combinations, and we did not assess the appropriateness of use. It is possible that sicker patients are housed in rooms closer to the nurses’ station, creating connections between them. Given the high volume at our hospital, most beds are assigned as soon as they become available, with less prioritization based on acuity. We included all hospitalized patients and averaged the nonpatient features for the last 7 days of hospitalization for patients who did not develop HOB. A case-control study, which would have matched patients with and without HOB based on the duration of hospitalization to balance the time at risk for developing HOB, would have drastically reduced the number of patients included and thus significantly limited the number of examined features. While we tried to include all potential features associated with HOB in our causal models, residual confounding may have been missed.

## Conclusions

In this prognostic study using data from 52 442 patients, we found that nonpatient features improved HOB predictive models. Our findings suggest that the mean number of HCWs per day and administration of antipseudomonal antibiotics in prior occupants were associated with HOB, highlighting the importance of antimicrobial stewardship efforts to curb the use of broad-spectrum antibiotics when unnecessary.

## Notes

Supplement 1. **eTable 1.** Data collection and preprocessing **eTable 2.** Cohort characteristics for MRSA HOB and patients without MRSA HOB

Supplement 2.
Data Sharing Statement
