# Error in Byline

**DOI:** 10.1001/jamanetworkopen.2025.30284

**Published:** 2025-08-06

In the Original Investigation titled “Integrating Nonindividual Patient Features in Machine Learning Models of Hospital-Onset Bacteremia,”^1^ published July 2, 2025, in the byline, the middle initial was missing from author David K. Warren’s name. This article has been corrected.^1^
