# Supplementary material for: Integrating Nonindividual Patient Features in Machine Learning Models of Hospital-Onset Bacteremia
Source: JAMA Netw Open. 2025 Jul 2;8(7):e2518815. doi: 10.1001/jamanetworkopen.2025.18815 (PMC12223889; doi:10.1001/jamanetworkopen.2025.18815)
Supplement: Supplement 1. — eTable 1. Data collection and preprocessing eTable 2. Cohort characteristics for MRSA HOB and patients without MRSA HOB [file jamanetwopen-e2518815-s001.pdf]

Supplemental Online Content

Vazquez Guillamet MC, Zhang J, Bewley A, et al. Integrating nonindividual patient features in machine learning models of hospital-onset bacteremia. *JAMA Netw Open*. 2025;8(7):e2518815. doi:10.1001/jamanetworkopen.2025.18815

eTable 1. Data collection and preprocessing

eTable 2. Cohort characteristics for methicillin resistant S. aureus (MRSA) hospital-onset bacteremia (HOB) and patients without MRSA HOB

This supplemental material has been provided by the authors to give readers additional information about their work.

© 2025 Vazquez Guillamet MC et al. *JAMA Network Open*.

eTable 1. Data collection and preprocessing

Feature	Description	Data Type
**Demographics**		
Sex	Male/ Female	Binary
Age	In years	Continuous
**Comorbidities**	ICD-10 codes predating the index admission	List of 1553 binary variables
**Procedures**		
**Placement of central venous catheter**	Procedure code at least 48hrs prior to HOB	Binary
**Endotracheal intubation**	Procedure code at least 48hrs prior to HOB	Binary
**Severity of illness prior to HOB**		
Shock	Use of vasopressors (norepinephrine, vasopressin, phenylephrine, angiotensin II, epinephrine) at least 48 hours before HOB	Binary
Mechanical ventilation	Use of endotracheal intubation at least 48 hours before HOB	Binary
ICU stay	Physical location in any of the BJH ICU	Binary
Duration of stay		
ICU stay (hours)	The duration of ICU stay	Continuous
Hospitalization (hours)	The duration of the hospital stay	Continuous
**Antibiotic use**		
Beta-lactams	The usage of Beta-lactams at least 48 hours before HOB	Binary
Anti-pseudomonal beta-lactams	The usage of Anti-pseudomonal beta-lactams (cefepime, piperacillin-tazobactam, meropenem, imipenem, aztreonam) at least 48 hours before HOB	Binary
Other antibiotics (gram-positives only, anaerobes, atypical)	The usage of other antibiotics (not beta-lactams/ anti-pseudomonal beta-lactams) at least 48 hours before HOB	Binary
**Interactions with healthcare workforce (HCW)**		

© 2025 Vazquez Guillamet MC et al. *JAMA Network Open*.

Feature	Description	Data Type
Average number of HCW per day	Average number of HCW providing care per day for the 7 days prior to HOB	Continuous
**Non-patient features**		
Number of rooms	Number of rooms visited during index hospitalization prior to HOB	Ordinal
Duration of room stay (hours)	The average duration of stay in each room	Continuous
ED waiting time (hours)	The duration of time spent in the ED	Continuous
Shared room time (hours)	The duration sharing a room with another patient	Continuous
Shared rooms with patients on antibiotics	Shared a room with another patient on Beta-lactams/Anti-pseudomonal beta-lactams/other antibiotics/any subtype of antibiotics	List of 4 binary variables
Inhabiting a room after a patient on antibiotics	Stayed in a room right after a patient on Beta-lactams/Anti-pseudomonal beta-lactams/other antibiotics/any subtype of antibiotics	List of 4 binary variables
Shared HCW with patients on antibiotics	Shared the same HCW with another patient on Beta-lactams/Anti-pseudomonal beta-lactams/other antibiotics /any subtype of antibiotics	List of 4 binary variables
Colonization pressure	Used in the MRSA model: The average ratio of patients with MRSA in the same unit during the 14 days prior to HOB	Continuous

* All risk factors are computed prior to HOB for HOB patients; otherwise, they are computed prior to the discharge time. Total number of cultures 393,162; total number of room stay records 171,425; total number of ICD10 codes 1,054,357; total number of notes 3,921,217

Abbreviations: BJH, Barnes Jewish Hospital; ED, Emergency Department; HCW, healthcare workforce; HOB, hospital-onset bacteremia, ICU, intensive care unit; ICD, International Classification of Diseases;

© 2025 Vazquez Guillamet MC et al. *JAMA Network Open*.

eTable 2. Cohort characteristics for MRSA HOB and patients without MRSA HOB

Feature	MRSA HOB, Number (%)	Patients without MRSA HOB, Number (%)	p value
**Number of patients**	70	34785	NA
**Demographics**			
Age, media (IQR), years	58.5 (45.3, 68.8)	60 (44, 70)	0.74
Male sex	41 (58.6)	17208 (49.5)	0.16
**Comorbidities**			
End-stage renal disease	17 (24.3)	1617 (4.7)	0.001
COPD, emphysema, chronic bronchitis	13 (18.6)	5667 (16.4)	0.72
Liver cirrhosis	5 (7.1)	1719 (5)	0.57
Hematological malignancies	6 (8.6)	2676 (7.7)	0.96
Solid organ transplantation	3 (4.3)	1002 (2.9)	0.73
Bacterial pneumonia	6 (8.6)	491 (1.4)	0.001
History of sepsis	37 (52.9)	2627 (7.6)	0.001
GNB infections	5 (7.1)	1905 (5.5)	0.73
Resistance to antimicrobials	5 (7.1)	531 (1.5)	0.001
Central venous catheter	22 (31.4)	1807 (5.2)	0.001
**Severity of illness**			
Septic shock	32 (45.7)	3920 (11.3)	0.001
Mechanical ventilation	10 (14.3)	866 (2.5)	0.001
ICU stay prior to HOB	44 (62.9)	8279 (23.8)	0.001
Duration of ICU stay, median (IQR), hours	75.5 (0.0, 243)	0.0, (0.0, 0.0)	0.001
Duration of hospitalization prior to HOB, median (IQR), hours	340.8 (162.2, 818.3)	147.7 (101.2, 249.4)	0.001
**Antibiotic use**			
Beta-lactams	46 (65.7)	14909 (42.9)	0.001

© 2025 Vazquez Guillamet MC et al. *JAMA Network Open*.

Feature	MRSA HOB, Number (%)	Patients without MRSA HOB, Number (%)	p value
Anti-pseudomonal beta-lactams	41 (58.6)	10903 (31.3)	0.001
Other antibiotics	49 (70)	14906 (42.9)	0.001
**Hospital features**			
Average number of HCW per day for the 7 days prior to HOB, mean (+/-2SD)	7.8 (5.1, 9.7)	6.4, (5.5, 7.6)	0.001
Number of rooms inhabited prior to HOB, median (IQR)	3 (2, 4)	2 (2, 3)	0.001
ER wait times, median (IQR), hours	0.00, 0.00, 5.48	0.00, 0.00, 7.46	0.08
**Direct/ indirect interaction with other patients**			
Duration of room sharing in the 7 days prior to HOB, mean (+/-2SD)	0.0 (0.0, 0.0)	34.2 (0.0, 153.7)	0.001
Shared rooms with patients colonized/ infected with MRSA	0 (0.0)	283 (0.8)	0.93
Inhabiting a room after a patient colonized/ infected with MRSA	4 (5.7)	1216 (3.5)	0.49
Shared HCW with patients colonized/ infected with MRSA	69 (98.6)	29614 (85.1)	0.001
Colonization pressure, mean (+/-2SD)	0.06 (0.02, 0.1)	0.01 (0.0, 0.04)	0.001

Abbreviations: COPD, chronic obstructive pulmonary disease; ED, emergency department; HCW, healthcare workforce; HOB, hospital-onset bacteremia; ICU, intensive care unit; IQR, interquartile range; MRSA, methicillin-resistant *S. aureus*; SD, standard deviation.

© 2025 Vazquez Guillamet MC et al. *JAMA Network Open*.
