# Supplementary material for: Integrating Nonindividual Patient Features in Machine Learning Models of Hospital-Onset Bacteremia
Source: JAMA Netw Open. 2025 Jul 2;8(7):e2518815. doi: 10.1001/jamanetworkopen.2025.18815 (PMC12223889; doi:10.1001/jamanetworkopen.2025.18815)
Supplement: Supplement 2. — Data Sharing Statement [file jamanetwopen-e2518815-s002.pdf]

Data Sharing Statement

Vazquez-Guillamet. Integrating Nonindividual Patient Features in Machine Learning Models Of Hospital-Onset Bacteremia. *JAMA Netw Open*. Published July 02, 2025. doi:10.1001/jamanetworkopen.2025.18815

Data
Data available: Yes
Data types: Deidentified participant data, Data dictionary, Other (please specify)
Additional Information: Analytic code
How to access data: Analytic code is available at https://github.com/afbewley/hospital-onset_bacteremia. The de-identified data will be available in the Washington University's medical repository after project approval and data user agreements.
When available: beginning date: 06-01-2025, end date: 05-31-2034

Supporting Documents
Document types: Statistical/analytic code
How to access documents: Analytic code is available at https://github.com/afbewley/hospital-onset_bacteremia.
When available: With publication

Additional Information
Who can access the data: researchers whose proposed use of the data has been approved
Types of analyses: de-dentified data
Mechanisms of data availability: after approval of proposal and with a signed DUA in place
